# Development and Internal Evaluation of AI-Assisted Cervical Muscle-Based Scores (FUNC-RISK) in Head and Neck Cancer: A Pilot Study

**DOI:** 10.3390/cancers17243968

**Published:** 2025-12-12

**Authors:** Laura Ferrera-Alayón, Fiorella Ximena Palmas-Candia, Barbara Salas-Salas, Jesús María González-Martín, Raquel Diaz-Saavedra, Anais Ramos-Ortiz, Pedro C. Lara, Marta Lloret Sáez-Bravo

**Affiliations:** 1 Department of Clinical Sciences, University of Las Palmas de Gran Canaria (ULPGC), 35010 Las Palmas de Gran Canaria, Spain; lferala@gobiernodecanarias.org; 2 Department of Radiation Oncology, University Hospital Dr Negrín, 35010 Las Palmas de Gran Canaria, Spain; 3 Endocrinology and Nutrition Department, Vall d’Hebron University Hospital, 08035 Barcelona, Spain; 4 Department of Medicine, Fernando Pessoa Canarias University, 35001 Las Palmas de Gran Canaria, Spain; 5 Unidad de investigación del HUGCDN, 35019 Las Palmas de Gran Canaria, Spain; 6 CIBER de Enfermedades Respiratorias, Instituto de Salud Carlos III, 28029 Madrid, Spain; 7 Canarian Comprehensive Cancer Center, San Roque University Hospital, 35010 Las Palmas de Gran Canaria, Spain; 8 Canarian Institute for Cancer Research (FICIC), 38320 La Laguna, Spain

**Keywords:** artificial intelligence, head and neck, cancer, radiotherapy, corporal composition

## Abstract

Background: An accurate prognostic assessment is essential to optimize treatment strategies in head and neck cancer (HNC). This study aimed to develop and internally evaluate an AI-assisted survival risk score derived from automatically quantified cervical muscle parameters on routine radiotherapy-planning CT scans. Methods: Pretreatment CT images were processed in a single-center cohort of 65 HNC patients, using AI-assisted automated segmentation to obtain the cervical skeletal muscle index (SMI), intramuscular adipose tissue area (IMAT), and mean muscle attenuation (HU). A multivariable Cox regression model was used to generate the continuous FUNC-RISK score, and model performance was assessed using time-dependent ROC curves at 36 and 60 months. Results: Patient-, tumor-, and treatment-related characteristics were not predictive of survival. SMI (*p* = 0.006) and IMAT (*p* = 0.047) were significantly associated with overall survival in a univariable analysis, while HU showed a borderline association (*p* = 0.087). All three parameters were included in the multivariable model, yielding the following equation: FUNC-RISK = (−0.364 × SMI) + (−0.087 × IMAT) + (0.011 × HU). The model demonstrated moderate discrimination (AUC = 0.734 at 36 months; 95% CI 0.604–0.863; *p* = 0.002, and AUC = 0.689 at 60 months; 95% CI 0.558–0.819; *p* = 0.009). Based on the median score (−3.18), patients were stratified into low- and high-risk groups. Five-year overall survival was 71.9% ± 7.9% for the low-risk group versus 39.4% ± 8.5% for the high-risk group (*p* = 0.006). Conclusions: FUNC-RISK provides preliminary evidence of clinically meaningful prognostic stratification based on AI-derived cervical muscle quantity and quality metrics obtained from routine radiotherapy-planning CT scans. These exploratory results support the potential role of automated body-composition analysis in personalized risk assessment for HNC, although external multicenter validation is required before clinical implementation.

## Simple Summary

This study aimed to develop and internally evaluate an AI-assisted survival risk score based on automatically quantified cervical muscle parameters. It was conducted in a single-center cohort of 65 patients with head and neck cancer (HNC). AI-powered segmentation of planning CT scans enabled automated extraction of the cervical skeletal muscle index (SMI), an intramuscular adipose tissue area (IMAT) and mean muscle attenuation expressed in Hounsfield units (HUs). The resulting FUNC-RISK score showed significant prognostic discrimination, with 5-year overall survival rates of 71.9% ± 7.9% in the low-risk group and 39.4% ± 8.5% in the high-risk group (*p* = 0.006). FUNC-RISK offers clinically meaningful stratification based on AI-derived muscle quantity and quality metrics from routine radiotherapy CT scans. These results support the potential of automated CT-based body-composition analysis to enhance personalized prognostic assessment in head and neck oncology.

## 1. Introduction

Head and neck cancer (HNC) is the seventh most prevalent malignancy worldwide, with an estimated number of 890,000 new cases and 450,000 deaths each year, underscoring its considerable global burden [1,2]. Despite therapeutic advances in surgery, radiotherapy (RT) and systemic therapies, survival outcomes remain suboptimal, particularly in locally advanced disease [3,4,5]. These limitations highlight the need for more refined prognostic tools to identify vulnerable patients and optimize therapeutic decision-making.

Beyond tumor stage and treatment modalities, growing evidence indicates that host-related factors—nutritional, metabolic and functional—play a decisive role in treatment tolerance and survival in HNC [3,4,5,6,7]. Sarcopenia and impaired muscle quality have emerged as key prognostic markers. Reduced muscle mass, lower muscle attenuation and greater fat infiltration are consistently associated with increased toxicity, treatment interruptions and poorer outcomes in patients receiving chemoradiotherapy [5,6,7,8,9,10,11,12]. These alterations reflect complex interactions between nutritional decline, systemic inflammation and metabolic dysregulation that influence both short- and long-term prognosis [6,10,11,12,13].

Computed tomography (CT) is the reference method for body-composition assessment. It enables precise quantification of muscle area and attenuation, using standardized Hounsfield Unit (HU) thresholds [10,11,14]. Although, traditionally, muscle evaluation relies on measurements at the L3 vertebral level, patients with HNC rarely undergo abdominal imaging. For this reason, measurements at the third cervical vertebra (C3) have been validated as reliable surrogates, demonstrating strong correlation with L3-derived metrics, and retaining prognostic significance in HNC cohorts [7,9,14,15,16,17]. Importantly, these parameters can be obtained directly from routine RT-planning CT scans, avoiding additional imaging or radiation exposure [13,16,17,18].

Despite increasing interest in body-composition analysis, many published studies rely on manual or semi-automated segmentation, which is time-consuming. It also introduces inter-observer variability, limiting its clinical applicability [6,10,16,17]. This methodological heterogeneity has impeded the standardization and establishment of widely accepted cut-offs for sarcopenia or myosteatosis [16,17,19,20]. Recent advances in artificial intelligence (AI) allow rapid, reproducible and observer-independent segmentation across large datasets, enabling standardized extraction of muscle area, adipose tissue and attenuation from CT scans [13,14,15,16,19,20,21]. These developments provide an opportunity to integrate objective, automated morphometric biomarkers into routine RT workflows, bridging the gap between quantitative imaging and clinical outcomes in HNC [7,8,9,13,14,18,20,22].

The aim of this study was to develop and internally evaluate a fully automated prognostic score (FUNC-RISK) integrating cervical skeletal muscle index (SMI), intramuscular adipose tissue (IMAT), and muscle attenuation (HU) derived from AI-based analysis of RT-planning CT scans. The model was designed to evaluate its ability to predict overall survival (OS) at 36 and 60 months, and to explore its potential clinical applicability as an objective, reproducible and scalable tool in radiation oncology.

## 2. Materials and Methods

### 2.1. Study Design and Patient Selection

The patient cohort analyzed in this prospective study has been previously described in detail in two related publications from our group [21,23]. In summary, we included adults aged ≥ 18 years with histologically confirmed squamous cell carcinoma of the head and neck, who were referred for curative-intent RT at the Department of Radiation Oncology, University Hospital Dr. Negrín (Las Palmas, Spain). Patients receiving concurrent systemic therapy were also eligible. Exclusion criteria were age < 18 years, non-squamous histology, palliative RT indication and cervical lymph node metastases from an unknown primary tumor. Oropharyngeal squamous cell carcinomas were not included in this cohort; therefore, HPV/p16 status was not applicable.

From the 68 evaluable patients reported in our earlier publication [21,23], three were subsequently excluded because they had cervical metastases from an unknown primary site. The final cohort consisted of 65 patients, with complete imaging and clinical data suitable for automated analysis and survival assessment.

The study protocol was approved by the institutional Ethics Committee (CEIm 2022-377-1), and all participants provided written informed consent before inclusion and treatment. The study procedures were aligned with our previously reported research on dysphagia screening and AI-based muscle composition analysis in HNC [21,23].

All RT-planning CT scans were acquired using a thermoplastic head-and-shoulder mask and an individualized headrest to ensure reproducible immobilization in a neutral position. This setup minimized anatomical variability and avoided angular deviations that could interfere with the assessment of cervical muscle parameters, such as CSA C3 and SMI C3.

### 2.2. Treatment, Recruitment Period and Follow-Up

All patients received curative-intent external-beam RT with IMRT/VMAT and daily image guidance, according to institutional protocols. Target volumes were contoured per international guidelines, and treatment plans were generated from the simulation CT described above.

For definitive RT, the prescribed dose was 70 Gy in 35 fractions (2 Gy/fx) to the gross tumor volume and 63–66 Gy to high-risk subclinical regions. For postoperative adjuvant RT, total doses of 63–66 Gy were delivered depending on pathological risk factors.

Concurrent systemic therapy was administered in 64.6% of patients, consisting of cisplatin-based chemotherapy (43.1%) or cetuximab (21.5%), depending on institutional standards and patient eligibility.

The recruitment period extended from November 2019 to January 2021, including all consecutive eligible patients treated at the Department of Radiation Oncology, University Hospital of Gran Canaria Dr. Negrín (Las Palmas, Spain).

The index date for survival analyses was the start of RT. The primary endpoint was OS, defined as the time from RT initiation to death from any cause. Patients alive at the data lock were censored at the date of last documented follow-up.

Follow-up was conducted according to institutional policy: clinical assessments during RT, at 4–6 weeks after treatment, every 3–4 months during the first 2 years and every 6–12 months thereafter. Vital status and dates of last contact were obtained from electronic medical records and hospital administrative databases.

### 2.3. Image Acquisition and Body Composition Analysis

All patients underwent planning CT scans acquired in treatment position as part of the standard RT workflow. No additional imaging or radiation exposure was required, as all analyses were performed on these scans. Images were acquired using a Siemens Somatom scanner (Siemens Healthineers, Erlangen, Germany). The scan range extended from the cranial vertex to the carina, ensuring consistent coverage of the cervical musculature at the C3 level.

CT slices were processed using the AI-based segmentation software FocusedON 2.1.0. (ARTIS, Las Palmas de Gran Canaria, Spain; https://focusedon.es, accessed on 5 December 2025). Skeletal muscle was identified using a predefined HU window of −29 to +150, and tissue-specific cross-sectional areas (cm^2^) were derived from voxel counts and pixel dimensions. The resulting metrics—SMI (cm^2^/m^2^), IMAT (cm^2^), and HU—were exported in tabular format for statistical analysis.

AI-based segmentation at the C3 level was generated by a custom 3D U-Net architecture integrated into FocusedON 2.1.0. To ensure anatomical accuracy, a human-in-the-loop workflow was applied, every axial slice around C3 was visually inspected, and manual corrections were performed when needed by a trained radiation oncologist. The final segmentation masks, therefore, corresponded to expert-validated contours equivalent to manual ground-truth segmentation. The effective segmentation failure rate was zero, because any mismatch was detected and corrected before data extraction. Standardized RT-simulation imaging minimized inter-scan variability, and mandatory human review prevented any AI-related inconsistencies from propagating into the dataset.

### 2.4. Model Development and Statistical Analysis

Statistical analyses were conducted using IBM SPSS Statistics, version 26 (IBM Corp., Armonk, NY, USA). Continuous variables were described using mean ± standard deviation (SD) or median and interquartile range (IQR), depending on data distribution. Categorical variables were reported as counts and percentages. The Kolmogorov–Smirnov test was applied to evaluate the assumption of normality.

Univariable Cox proportional hazards regression analyses were first performed to examine the association between patient, treatment and imaging-derived variables—SMI, IMAT and HU—and OS. Variables with *p* < 0.10 in univariable analysis were subsequently entered into a multivariable Cox regression model (enter method) to obtain adjusted hazard ratios (HRs) with 95% confidence intervals (CIs).

A composite survival risk score, termed FUNC-RISK, was derived post hoc from the regression coefficients of the final multivariable model, reflecting the combined prognostic contribution of muscle quantity and quality parameters. Model discrimination was evaluated using time-dependent receiver operating characteristic (ROC) curves, with calculation of the area under the curve (AUC) at 36 and 60 months. For clinical interpretability, patients were dichotomized into low- and high-risk groups, based on the median FUNC-RISK value.

OS was estimated using the Kaplan–Meier method and compared with the log-rank test. Youden’s index was explored to identify alternative thresholds for FUNC-RISK, reporting corresponding sensitivity and specificity. All statistical tests were two-sided, with *p* < 0.05 considered statistically significant.

As an internal assessment of discrimination stability, Harrell’s concordance index (C-index) was calculated for the FUNC-RISK score, using OS time and event status. A non-parametric bootstrap procedure with 500 resamples was applied to estimate the variability of the C-index. Additionally, as an exploratory sensitivity analysis, the cohort was randomly partitioned into five folds. The C-index was recalculated within each fold to explore the consistency of FUNC-RISK performance across different patient subsets.

Continuous variables were retained in their natural units to preserve clinical interpretability. Multicollinearity among SMI, IMAT and HU was assessed, using pairwise correlations and variance inflation factors (all < 2), confirming absence of problematic collinearity. Given the exploratory pilot nature of the study and the limited number of events, a conventional Cox model was favored over penalized regression approaches (e.g., lasso/ridge). This type of approach requires larger datasets and would reduce interpretability of the resulting score.

## 3. Results

### 3.1. Patient’s Characteristics

**Figure 1:**
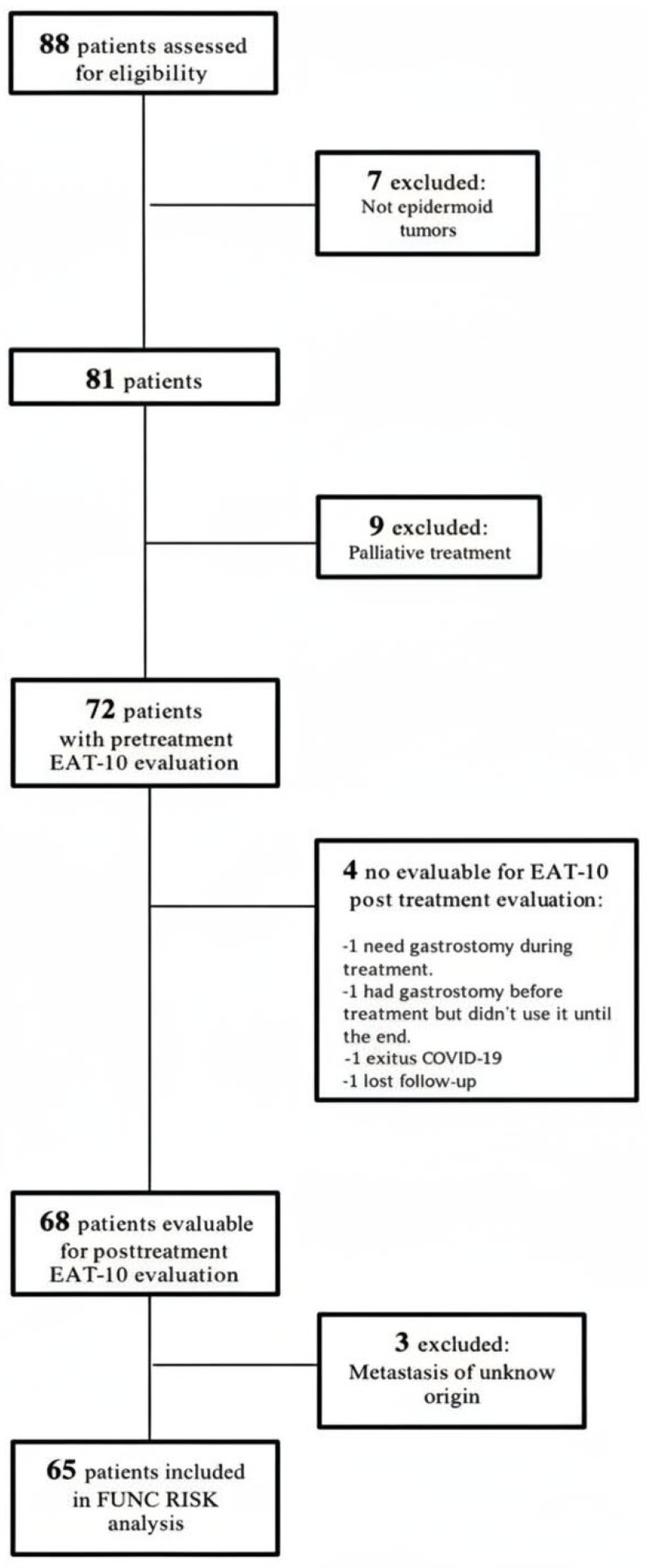
Patient’s flowchart.

A total of 65 patients with complete imaging and clinical data were included in the present analysis. The characteristics of this prospective cohort have been described in detail in earlier publications [21,23]. The patient selection process is summarized in Figure 1, and the main demographic and clinical characteristics are presented in Table 1. The mean age at diagnosis was 68.8 ± 10.4 years (range 48–90).

All patients had histologically confirmed squamous cell carcinoma of the head and neck and were treated with curative intent, receiving either definitive RT (56.9%) or postoperative adjuvant RT following surgery (43.1%), with or without concurrent systemic therapy. The majority presented with locally advanced disease (stages III–IV, 72.3%) and received concurrent systemic treatment (64.6%). At the data lock, in July 2025, the median follow-up among survivors was 57 months.

Baseline body-composition parameters obtained from pretreatment planning CTs at the C3 level are summarized in Table 2. Quantitative body-composition metrics were automatically extracted using the AI-assisted CT-segmentation software FocusedON 2.1.0. This tool provided consistent and reproducible measurements of muscle area, fat infiltration and muscle attenuation.

### 3.2. Survival Analysis and Model Performance

During follow-up, 29 deaths (44.6%) were recorded among the 65 patients included in the study. In the univariable analysis, none of the patient-, tumor- and treatment-related variables showed a statistically significant association with OS (Table 1).

Regarding the IA-assisted body composition analysis, the SMI and IMAT were associated with improved OS, while HU showed a statistical trend. In the multivariable Cox regression model, including all three variables, SMI and IMAT remained independently associated with OS, whereas HU did not reach statistical significance.

Results from both univariate and multivariable Cox regression analyses are presented in Table 3. Variables derived from automated AI-based quantification at the C3 level. HRs < 1 represent protective effects. The multivariable model includes all three parameters entered simultaneously.

The β-coefficients obtained from the final multivariable model were used to generate a continuous composite risk score, termed FUNC-RISK. Each coefficient reflects the relative contribution of its variable to OS: negative values indicate a protective effect, whereas positive values indicate a higher mortality risk. These coefficients, directly derived from the multivariable Cox model shown in Table 3, were combined to compute the individualized survival risk score. FUNC-RISK was calculated using the following equation:

FUNC RISK = (−0.364 × SMI [cm^2^/m^2^]) + (−0.087 × IMAT [cm^2^]) + (0.011 × HU [Hounsfield Units]) (1)

Higher FUNC-RISK values indicate increased mortality risk. When applied to our patient cohort, FUNC-RISK scores ranged from −4.67 to −1.34, with a median of −3.18 (mean = −3.10 ± 0.71). In univariable Cox regression, the FUNC-RISK score was significantly associated with OS (HR = 2.69, 95% CI 1.48–4.90, *p* = 0.001), confirming that higher FUNC-RISK values predicted poorer outcomes. Patients were then stratified according to the median FUNC-RISK value, defining low-risk (*n* = 32) and high-risk (*n* = 33) groups. Baseline characteristics according to these groups are summarized in Table 4. Categorical variables were compared using χ^2^ or Fisher’s exact test, and continuous variables using the Mann–Whitney U test. A *p* < 0.05 was considered statistically significant. “Other sites” include nasopharynx, maxillary sinus and salivary gland tumors.

No statistically significant differences were observed between groups except for sex (*p* = 0.044). 23 out of 32 low-risk patients (71.9%) were alive at the follow-up lock compared with 13 out of 33 high-risk patients (39.4%) (*p* = 0.008).

**Figure 2:**
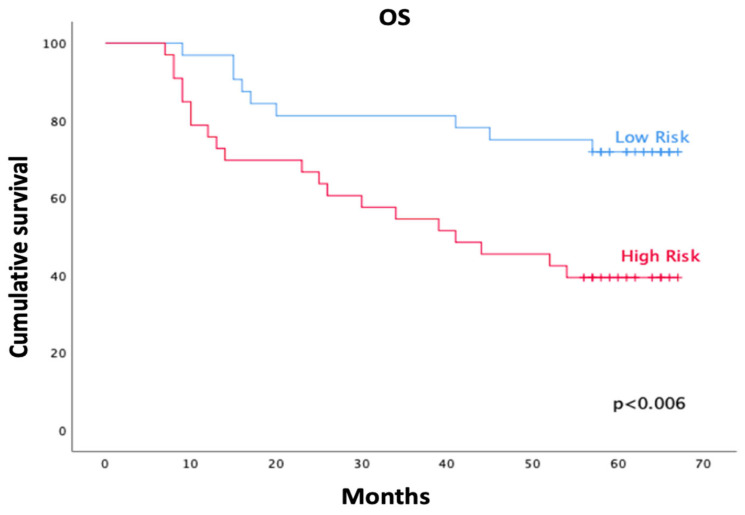
Kaplan–Meier curves of overall survival (OS) according to FUNC-RISK groups. The low-risk group (blue) showed significantly higher survival than the high-risk group (red) (log-rank χ^2^ = 7.51; *p* = 0.006).

Kaplan–Meier analysis showed significantly lower OS in the high-risk group compared with the low-risk group (log-rank χ^2^ = 7.51; *p* = 0.006). At five years, actuarial OS was 71.9% ± 7.9% (95% CI 0.56–0.86) in the low-risk group and 39.4% ± 8.5% (95% CI 0.23–0.55) in the high-risk group (Figure 2). Mean survival time was 54.1 months (95% CI 46.7–61.4) in the low-risk group and 41.6 months (95% CI 33.2–49.9) in the high-risk group.

The median OS was reached only in the high-risk group (41.0 months; 95% CI 16.1–65.9), whereas median survival was not reached among low-risk patients at the time of analysis.

### 3.3. Model Discrimination (ROC Curves)

The discriminatory ability of the FUNC-RISK model was evaluated using time-dependent ROC curves for OS prediction. The model achieved an AUC of 0.734 at 36 months (95% CI 0.604–0.863; *p* = 0.002) and 0.689 at 60 months (95% CI 0.558–0.819; *p* = 0.009), indicating moderate prognostic discrimination.

**Figure 3:**
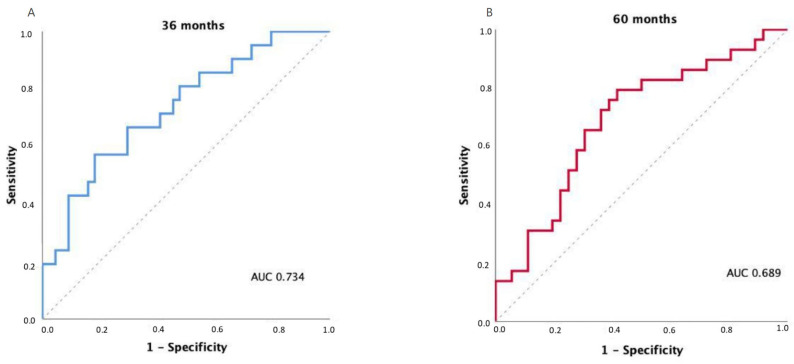
Time-dependent receiver operating characteristic (ROC) curves for OS prediction using the FUNC-RISK model at (**A**) 36 months and (**B**) 60 months.

Exploratory analyses using Youden’s index identified an optimal FUNC-RISK threshold of –3.2962 at both 36 and 60 months, corresponding to sensitivities of 0.81 and 0.79 and specificities of 0.50 and 0.56, respectively. The model showed comparable discrimination across both time points, as illustrated in Figure 3.

These results indicate that FUNC-RISK provides consistent discrimination across time, maintaining its prognostic accuracy over medium and long-term follow-up.

In terms of global discrimination, the FUNC-RISK score achieved a Harrell’s C-index of 0.67 for OS. Internal bootstrap validation (500 resamples) produced a mean C-index of 0.68 (approximate 95% bootstrap interval: 0.57–0.78), indicating moderate discrimination with expected uncertainty, given the sample size. In an exploratory five-fold resampling analysis, performance remained comparable across subsets (mean C-index 0.73; range 0.53–0.83), further supporting the internal stability of the score within this pilot cohort.

## 4. Discussion

This study developed and internally evaluated, for the first time, an AI-assisted survival risk score (FUNC-RISK) integrating cervical muscle quantity and quality parameters in patients with HNC treated with curative-intent RT. The model demonstrated moderate but consistent discrimination for OS (AUC = 0.734 at 36 months; 0.689 at 60 months), and survival differed significantly between the low- and high-risk groups (*p* = 0.006), supporting its potential clinical applicability. At five years, OS was 71.9% in the low-risk group versus 39.4% in the high-risk group. The median OS was not reached among low-risk patients, while it was 41.0 months in the high-risk group. Together, these findings reinforce the prognostic relevance of body-composition metrics derived from RT-planning CT scans. They also highlight their feasibility for integration into risk stratification frameworks in radiation oncology.

Compared with previously published AI-based tools—which have primarily focused on diagnosing sarcopenia or automating muscle quantification—FUNC-RISK introduces several distinctive elements. First, it integrates three complementary cervical-level body-composition parameters (SMI, IMAT, and HU) into a single multivariable prognostic equation, specifically designed to predict OS rather than classify sarcopenia. Second, unlike most existing models derived from abdominal (L3) imaging, FUNC-RISK is based exclusively on C3-level measurements obtained from routine RT-planning CT scans, allowing universal applicability in HNC without requiring additional imaging. Third, the model relies on fully automated, clinically implemented segmentation, rather than research-based algorithms, ensuring reproducibility and immediate clinical usability. Finally, FUNC-RISK was intentionally developed without incorporating clinical or tumor-related variables, which enabled us to assess the prognostic value of muscle quantity and quality independently from other established determinants of survival.

Given the modest sample size and the exploratory nature of this pilot model, the HRs obtained from the multivariable Cox analysis should be interpreted with caution. Although the coefficients reflect the independent contribution of each imaging-derived parameter within this cohort, statistical variability and potential model optimism cannot be excluded. The internal discrimination analyses (bootstrap and five-fold resampling) further support this interpretation, showing moderate but variable performance across subsets. Therefore, the HR estimates should be considered hypothesis-generating until validated in larger independent datasets.

The selection of SMI, IMAT and HU was based on biological plausibility. SMI captures overall muscle quantity. IMAT reflects intramuscular fat infiltration, associated with metabolic and inflammatory alterations, and HU represents muscle quality through tissue density. Because each parameter describes a distinct dimension of muscle structure and function, their combined use provides a more comprehensive assessment of muscle health and of the prognostic relevance of these muscle characteristics in patients with head and neck cancer.

In practical terms, FUNC-RISK combines three cervical muscle parameters (SMI, IMAT and HU) into a single score that provides a direct estimate of survival risk using only the RT-planning CT scan. This allows clinicians to identify high-risk patients early and consider timely nutritional, metabolic or functional interventions before their treatment begins.

From a clinical perspective, the marked separation of the Kaplan–Meier curves indicates that high-risk patients experience earlier and more frequent mortality events, despite receiving similar oncologic treatments. This suggests that FUNC-RISK may help clinicians identify individuals who could benefit from closer surveillance, earlier nutritional/functional assessment and proactive supportive care. Conversely, the more favorable five-year survival observed in low-risk patients reflects a more resilient physiological reserve, which may help guide prognostic conversations with patients. The moderate AUC values at 36 and 60 months further support the use of FUNC-RISK as a complementary tool to assist risk-adapted counseling and individualized follow-up strategies in routine practice.

In everyday clinical practice, FUNC-RISK may help identify patients who could benefit from early supportive interventions. For example, a patient classified as high-risk based on low SMI, high IMAT and reduced HU could be referred for early nutritional counseling or physical conditioning before starting RT, with the aim of improving physiological reserve and treatment tolerance. Similarly, high-risk classification may prompt clinicians to schedule closer follow-up visits during and after treatment to monitor weight trends, functional status or overall recovery. Conversely, low-risk patients may be managed with standard surveillance schedules, as their cervical muscle profile reflects greater baseline resilience. These examples illustrate how FUNC-RISK could complement traditional clinical factors to support individualized care pathways in head and neck oncology.

When examining the distribution of FUNC-RISK categories across treatment modalities, both radical (*n* = 37) and adjuvant (*n* = 28) treatments included comparable proportions of low- and high-risk patients. Specifically, the radical cohort contained 19 low-risk and 18 high-risk patients, while the adjuvant cohort included 13 low-risk and 15 high-risk patients. No treatment modality showed a dominant clustering of either category. This balanced distribution suggests that FUNC-RISK provides prognostic information that is applicable across different therapeutic pathways in HNC.

### 4.1. Cervical Muscle Quantification and Prognostic Relevance

Cervical-level body-composition metrics derived from routine planning CT scans have emerged as reliable surrogates for lumbar muscle assessment. Multiple studies have demonstrated that cross-sectional muscle area and SMI measured at the C3 level strongly correlate with L3 metrics and predict treatment-related outcomes and survival in HNC [16,24,25,26,27,28]. These data validate cervical muscle estimation as a practical and accurate alternative when abdominal imaging is not available.

Low SMI has consistently been associated with impaired functional capacity, increased treatment-related toxicity and reduced survival in patients undergoing RT or chemoradiotherapy [6,8,9,18,24,29,30]. Our findings align with this evidence, reinforcing the relevance of pre-treatment muscle assessment as a prognostic biomarker.

Beyond muscle quantity, parameters of muscle quality—namely, mean attenuation in HU and IMAT—also demonstrate prognostic significance [3,8,18,24]. Myosteatosis, characterized by increased fat infiltration and reduced attenuation, is linked to systemic inflammation and metabolic dysregulation, and it has been associated with worse survival across cancer populations [3,18,24]. However, consistent with our observations, the relationship between IMAT and survival is not uniformly linear. Several cohorts have reported no association or even inverse trends, suggesting that IMAT may be influenced by patient phenotype, treatment exposures or regional muscle composition [18,24,30]. These discrepancies highlight the complex interaction between muscle quantity, quality and systemic metabolic state, underscoring the need for further mechanistic investigation. Collectively, current evidence supports cervical muscle metrics as biologically meaningful and independent prognostic markers in HNC.

Importantly, the FUNC-RISK model was intentionally developed using only imaging-derived muscle parameters to isolate their intrinsic prognostic value. This development was independent of conventional clinical variables such as stage, age or systemic therapy. Moreover, it minimizes potential confounding from tumor- or treatment-related factors and allows a focused evaluation of muscle quantity and quality, as survival determinants.

Although traditional prognostic variables remain essential in clinical decision-making, imaging-based models such as FUNC-RISK may provide complementary insight into physiological and metabolic reserve. Accordingly, the model should be interpreted as a biological complement—rather than a substitute—to established clinical predictors. Future research in larger multicenter cohorts should evaluate the incremental prognostic value of FUNC-RISK when combined with standard clinical variables.

### 4.2. Artificial Intelligence and Automated Segmentation

Traditional manual or semi-automatic segmentation methods are time-consuming and subject to interobserver variability, which limits reproducibility and hinders clinical translation [2,7,10,13,16,18]. Recent advances in deep-learning-based segmentation have substantially improved this processing time [10,11,14,16]. Automated pipelines trained on head-and-neck CT datasets have reported Dice similarity coefficients exceeding 0.95, supporting their robustness for quantifying SMI, IMAT, and HU [2,10,16,21]. These approaches allow rapid extraction of morphometric biomarkers directly from routine planning scans, without additional imaging, radiation exposure or workflow modifications [7,13,16,18,21].

Within this framework, the FUNC-RISK model represents a novel step toward integrating AI-derived body composition metrics into prognostic modeling. By combining quantity and composition of cervical muscle into a single continuous score, FUNC-RISK provides an objective, reproducible, fully automated imaging biomarker. It can also complement established clinicopathologic factors for risk stratification and adaptive decision-making [7,13,18,21,23]. This approach may facilitate the broader adoption of imaging-based prognostic tools in routine head and neck oncology practice.

### 4.3. Strengths

The primary strength of this study is the use of fully automated AI-based segmentation applied to routine RT-planning CT scans, enabling reproducible, objective, and observer-independent quantification of cervical muscle parameters. The exclusive analysis of pretreatment imaging avoids confounding related to treatment-induced anatomical or metabolic changes. In addition, the inclusion of a homogeneous cohort of HNC patients treated with curative-intent RT (definitive or postoperative), within a single institution, enhances internal validity.

An additional strength lies in the rigorous methodological framework used for model development. It incorporated multivariable Cox regression, time-dependent ROC analysis, and transparent reporting of model coefficients in order to ensure reproducibility and facilitate future external validation. Additionally, the combined evaluation of both muscle quantity (SMI) and muscle quality (HU and IMAT) extends beyond conventional sarcopenia assessment and offers a more comprehensive characterization of muscle health as a prognostic biomarker in HNC.

### 4.4. Limitations

This study has several limitations that need to be acknowledged. First, it was conducted at a single center, with a relatively modest sample size. Thus, it may restrict generalizability. External validation in independent and larger cohorts is required to confirm the robustness of the FUNC-RISK model. Although this study was conceived as an exploratory pilot analysis, methodological evaluations of pilot sample sizes indicate that prognostic pilot studies commonly include 20–40 participants, when the aim is parameter estimation rather than hypothesis testing [25]. Therefore, the present sample size aligns with accepted ranges for pilot investigations. Nonetheless, some degree of model optimism cannot be excluded, as suggested by the variability observed in the internal bootstrap and resampling analyses, further reinforcing the need for multicenter external validation. Likewise, the HRs derived from the multivariable model should be interpreted with caution, as the limited cohort size may introduce statistical instability despite the internal validation procedures.

In addition, although calibration is a key component of prognostic model evaluation, the present cohort (*n* = 65) included only 29 survival events. This number is insufficient to generate reliable calibration curves or Brier scores. As recommended for pilot prognostic studies, we therefore reported only discrimination metrics, acknowledging that calibration analyses will require larger external cohorts, with adequate statistical power. The absence of calibration assessment further reinforces the exploratory nature of this model and the need for multicenter validation.

Second, although the segmentation process was fully automated through AI-assisted software, minor residual errors in tissue labeling cannot be excluded. Third, body-composition changes during treatment were not evaluated, and only baseline CTs were considered. Longitudinal analyses could provide additional insight into dynamic risk assessment. Finally, clinical variables such as nutritional interventions, inflammatory markers or comorbidity indices were not integrated into the model. Incorporating them could enhance predictive accuracy in future studies.

Despite these limitations, the present study provides a methodological foundation that may support future development, as well as validation of automated prognostic tools in radiation oncology.

### 4.5. Clinical Implications and Future Directions

The clinical implementation of automated body-composition analysis has the potential to enhance risk assessment in radiation oncology. Since RT-planning CT scans are routinely acquired for all patients, integrating AI-based segmentation directly into existing workflows requires no additional imaging, radiation or cost. Automated extraction of cervical muscle parameters could provide real-time information on nutritional and functional status, complementing conventional clinical data and performance indices. In particular, the FUNC-RISK score—by combining muscle quantity and quality metrics into a single prognostic model—may help identify high-risk patients before treatment initiation. Thus, it could enable early nutritional, metabolic or physical interventions aimed at improving resilience and treatment tolerance.

From a precision-medicine perspective, models such as FUNC-RISK could contribute to adaptive and individualized RT. Automated stratification may support treatment personalization by linking functional imaging biomarkers to outcome prediction, potentially informing dose adaptation, concurrent therapy decisions, or closer clinical follow-up. Moreover, incorporating these image-based biomarkers into multidisciplinary management could improve patient selection for supportive care and for clinical trials that target cachexia and sarcopenia in oncology.

Future research should prioritize external and multicentric validation of the FUNC-RISK model across diverse populations and imaging platforms to confirm its generalizability. Integrating the score with other emerging biomarkers—such as systemic inflammation indexes, radiomic texture features or molecular profiles—may further refine prognostic accuracy. Ultimately, embedding AI-based body-composition analytics into hospital information systems and treatment-planning software could enable fully automated, patient-specific risk profiling in radiation oncology.

## 5. Conclusions

This pilot study presents the preliminary development and internal evaluation of FUNC-RISK, an automated CT-derived prognostic score integrating cervical muscle quantity and quality parameters in patients with HNC. FUNC-RISK demonstrated clinically meaningful risk stratification and moderate prognostic discrimination, supporting the relevance of cervical muscle metrics as complementary biomarkers in radiation oncology. However, these findings should be interpreted with caution, given the single-center design and limited sample size. Future research should focus on external validation in larger, multicenter cohorts and on assessing the added prognostic value of FUNC-RISK when combined with conventional clinical variables. Taken together, this work provides a foundation for the potential integration of automated muscle-based imaging biomarkers into personalized care pathways in HNC.

## Figures and Tables

**Table 1:** Patient and treatment characteristics and their association with overall survival in univariable analysis.

Characteristic	Patients (%)	HR	95% CI	*p*-Value
Age (years) (68.8 ± 10.4, range 48–90)		1.036	0.997–1.075	0.069
Sex		0.957	0.333–2.750	0.935
Male	55 (84.6%)			
Female	10 (15.4%)			
Tumoral stage		0.836	0.35–1.99	0.676
E.I–II	18 (27.7%)			
E.III–IV	47 (72.3%)			
Tumor location		—	—	0.182
Nasopharynx	3 (4.6%)			
Oral cavity	25 (38.5%)			
Hypopharynx	7 (10.8%)			
Larynx	26 (40%)			
Other *	4 (6.1%)			
Loco-regional treatment		0.720	0.29–1.80	0.466
Radiotherapy	37 (56.9%)			
Surgery + radiotherapy	28 (43.1%)			
Systemic treatment		1.011	0.47–2.18	0.977
No	23 (35.4%)			
Yes	42 (64.6%)			

* Other sites include squamous cell carcinomas of the maxillary sinus and major salivary glands.

**Table 2:** Body composition metrics derived from AI-based CT analysis. SD: standard deviation, IQR: interquartile range.

Parameter	Mean ± SD	Median (IQR)	Range
Cervical SMI (cm^2^/m^2^)	9.7 ± 1.9	9.6 (8.6–10.8)	5.4–13.5
IMAT (cm^2^)	31.6 ± 9.5	31.0 (24.0–38.0)	16.8–45.0
Mean muscle attenuation (HU)	37.9 ± 7.2	38.0 (33.0–43.0)	20.0–48.0

**Table 3:** Relation of IA-assisted body composition parameters and survival in univariate analysis.

Variable	HR	95% CI	*p*-Value
Univariable analysis			
SMI (cm^2^/m^2^)	0.715	0.563–0.909	0.006
IMAT (cm^2^)	0.929	0.864–0.999	0.047
Mean muscle attenuation (HU)	1.013	0.998–1.028	0.087
Multivariable analysis			
SMI (cm^2^/m^2^)	0.695	0.529–0.913	0.009
IMAT (cm^2^)	0.917	0.843–0.996	0.041
Mean muscle attenuation (HU)	1.011	0.996–1.027	0.144

**Table 4:** Comparison of demographic and clinical characteristics according to FUNC RISK group.

Characteristic	Low Risk (*n* = 32)	High Risk (*n* = 33)	*p*-Value
Age, median (range) [years]	68 (48–85)	70 (50–90)	0.121
Gender	7 (21.9%) females 25 (78.1%) males	3 (9.1%) females 30 (90.9%) males	0.044
Tumor site			0.295
Nasopharynx	1 (3.1%)	2 (6.1%)	
Oral cavity	14 (43.8%)	11 (33.3%)	
Hypopharynx	12 (37.5%)	14 (42.4%)	
Larynx	5 (15.6%)	2 (6.1%)	
Other sites	0 (0%)	4 (12.1%)	
Clinical stage			0.302
Stage I–II	7 (21.9%)	11 (33.3%)	
Stage III–IV	25 (78.1%)	22 (66.7%)	
Systemic therapy	No = 10 (31.3%) Yes = 22 (68.8%)	No = 13 (39.4%) Yes = 20 (60.6%)	0.492

## Data Availability

The data presented in this study are available upon request from the corresponding author due to ethical reasons.
